# Multivariable Analysis of the Carbon Footprint of a Branded Beef Supply Chain Using Individual Animal Data and Carcass Characteristics

**DOI:** 10.3390/ani16162498

**Published:** 2026-08-11

**Authors:** Riley O’Shannessy, Stephen Wiedemann

**Affiliations:** Integrity Ag Pty Ltd., 10 Neil Street, Toowoomba City, QLD 4350, Australia; stephen.wiedemann@integrityag.net.au

**Keywords:** beef cattle, life cycle assessment, greenhouse gas, carbon footprint, red meat, grass-fed, natural grain, southern Australia, sustainable agriculture

## Abstract

Globally, beef customers are seeking verified information regarding the carbon footprint (CF) of the products they buy. As a major supplier of premium grass-finished and natural grain beef supplying markets world-wide, JBS Southern Australia developed a certified Farm Assured (FA) program, launched in 2013, to provide quality beef from independently audited suppliers. This study conducted a life cycle assessment (LCA) with ‘cradle to farm gate’ and ‘cradle to processor gate’ boundaries, using two reference flows—(i) one kilogram (kg) of liveweight (LW) at the farm gate, and (ii) one kg of boxed beef at the processor gate—to assess the greenhouse gas (GHG) CF for beef produced in southern Australia. This study is the first to integrate individual animal carcass characteristics with brand level CF analysis at scale. This was achieved by developing a uniquely comprehensive dataset, with primary data supplied by 200 farms and individual animal data provided for 514,922 heads of cattle. The mean farm gate CF was 11.7 (standard deviation 0.4) kg carbon dioxide equivalent (CO_2_-e) kg^−1^ LW, and the mean boxed beef CF was 24.1 kg CO_2_-e kg^−1^ boxed beef. The study’s novel approach to data collection allowed for the CF to be stratified by region, carcass characteristics, farm of origin and product brand. Analysis revealed that the lowest farm-average and individual animal CFs were 29% and 48% lower than the supply chain average, respectively. These findings indicate that the CFs of beef produced from grass and natural grain-finished production systems in southern Australia were comparable or lower than the CFs of beef entering similar markets.

## Simple Summary

Beef production is a significant source of greenhouse gas emissions, but current data are not detailed enough to connect emissions to specific products, brands, or farms. This study measured the carbon footprint of beef from a large, branded supply chain in southern Australia, sourcing from approximately 1600 independently audited farms raising grass-fed and natural grain-finished cattle. Using data from individual records for 514,922 cattle, 200 farm surveys (covering 11% of total supply and 19% of mean annual throughput) and regionally parameterised datasets, this study provides novel insights into the link between individual animal characteristics—such as weight and age at slaughter—and carbon footprint results at a branded product level across a large number of animals. The average carbon footprint was 11.7 kg of carbon dioxide equivalents per kilogram of live animal weight leaving the farm, and 24.1 kg of carbon dioxide equivalents per kilogram of boxed beef (the final packaged product). The best performing farms and individual animals had carbon footprints 29% and 48% below the supply chain average, respectively. Heavier, younger cattle at slaughter produced lower emissions per unit of beef. For context, these results were comparable to or lower than beef from other major trading nations. The findings show that improving cattle productivity can reduce the environmental footprint of beef, informing producers, brands, and consumers seeking lower-emission products.

## 1. Introduction

Global customers in premium beef markets are increasingly interested in understanding the carbon footprint (CF) of products they buy [1,2]. Beef CFs vary significantly between production systems [3]. To the authors’ knowledge, there are no published studies which have explored the difference in CF between production systems specific to a branded product, despite some beef products being supplied from specific production systems. Such brands include the JBS Southern Australia ‘Great Southern’ brand and the JBS Southern Australia ‘Portoro’ brand, which source products from cattle that are finished on natural grains.

There are few large beef-exporting nations in the world that have a lifetime traceability of cattle production for individual animals [4,5]. Recently, small-scale life cycle assessment (LCA) studies have assessed impacts using individual animal data, but this has been limited to the small datasets of McAuliffe et al. [6] (90 heads) and Gallo et al. [7] (564 heads) from research farms. These studies focused on primary production and did not link the individual animal data through to the beef product. Studies that have investigated the CF of ruminant protein at a product level have either used a small (<5) sample of case study farms [8], idealised data [9], or data that were not specifically connected to the product through lifetime traceability [10,11]. There is a gap in research that links primary production systems to brands at a large scale utilising livestock traceability data at a large scale. This gap limits the knowledge available to brands and supply chains with respect to the drivers of CFs and the variance in impacts at the producer level and for animal cohorts.

The present study assessed the CF of a large-scale branded beef supply chain using a sample of primary, on-farm data and a supply chain parameterised model, with individual animal data from all cattle in the program. This system allowed for a large-scale assessment linking production systems to brands, enabling a cohort-level analysis of the drivers of variance in CF using individual animal data. The unique dataset and model design enabled the stratification of livestock based on characteristics where brand attributes and CFs intersect, including weight and age at slaughter. Furthermore, the study provided novel insights into the variations within and between products, farms, and production regions. In this study, LCA was conducted using two functional units, each quantified by a reference flow: (i) one kilogram (kg) of liveweight (LW) at the farm gate, and (ii) one kg of boxed beef (including all edible portions of retail meat and edible offal) at the processor gate. These units were used to quantify and present the CF results throughout the analysis, allowing for the assessment of impacts at different stages of the supply chain.

Emissions from beef production are predominantly generated from primary production, where the CF is influenced by the efficiency of the breeding herd [12] and potentially land management. The quantum of emissions is reasonably well understood globally, based on the review of de Vries et al. [13] and global results from the Global Livestock Environmental Assessment (GLEAM) model [14]. However, with increased market scrutiny, customers are seeking information that is temporally and spatially specific to the products they buy. With common frameworks such as the Science Based Targets initiative (SBTi) requiring near-term Forest, Land and Agriculture (FLAG) emission intensity reductions of 2.4% per annum for beef (kg CO_2_-e kg^−1^ product) relative to a company-selected base year no earlier than 2015 and covering scope 1 and scope 3 FLAG emissions to the farm gate [15], small differences in baseline CF are highly relevant for informing emission reduction programs. Some information exists comparing production from grain and grass-finishing [16,17], but these did not examine production from southern Australia. In the southern, temperate regions of Australia, high-quality beef can be produced from natural grass-finishing systems where cattle graze year-round on pasture. JBS Southern Australia has developed a third-party-audited ‘Farm Assurance’ (FA) program to ensure that these practices are used for all cattle supplied to their Great Southern (GS) program, and has operated this program since 2013, covering approximately 1600 beef suppliers (confidential slaughter data provided). The JBS Farm Assured program requires third-party audits of on-farm practices to ensure that cattle are grass-fed and free-range, with no added antibiotics, hormones or GMOs in their feed. To be qualified as grass-fed, livestock must be continually grazed on a pasture-based system for their lifetime. Animals are not to be fed grain or grain by-products. In 2021 the company commissioned research to expand the program to include CF assessment. This represents a large-scale implementation of carbon footprinting in a complex supply chain. In addition to the grass-fed supply chain, the company has developed a specialist natural grain-finishing program. In this program, cattle are fed diets without antibiotics or ionophores, as defined in the JBS Farm Assurance Controlled Bovine–Beef, Natural Grain Standard [18]. The CFs of both production systems were unknown prior to the study.

The study was unique in the temporal, regional and production system specifics, together with the scale of implementation. Previous beef CF studies in Australia have used isolated farm case studies [19,20], idealised datasets [21], or regional statistics combined with a small number of farm datasets [12,22]. Similarly, global benchmarks such as the GLEAM program utilise regional statistics and global emission calculation methods [14], which have the effect of normalising results and reducing the specificity and insight that can be gained from the findings for a particular region and production system. Establishing a supply chain benchmark, and, in the future, measuring performance against this, requires primary data specific to the supply chain assessed. Having access to data from a large supply chain also provides a new opportunity for insights that have not been examined before in full cradle-to-gate LCA, such as the difference between beef brand programs and different combinations of age and weight of finished cattle. This study provided new insights on the relationship between these traditional beef market quality indicators and CF. Specifically, the study aimed to

1. Determine the baseline CF for an Australian grass-finished and natural grain supply chain, including specific branded beef products for the calendar year 2023 and a weighted average for calendar year 2023 covering all brands;
2. Provide a regional hotspot analysis of emission sources;
3. Examine short-term temporal trends in CF by investigating impacts across the calendar years 2020–2023.

## 2. Materials and Methods

### 2.1. System Boundaries and Reference Flow

**Figure 1:**
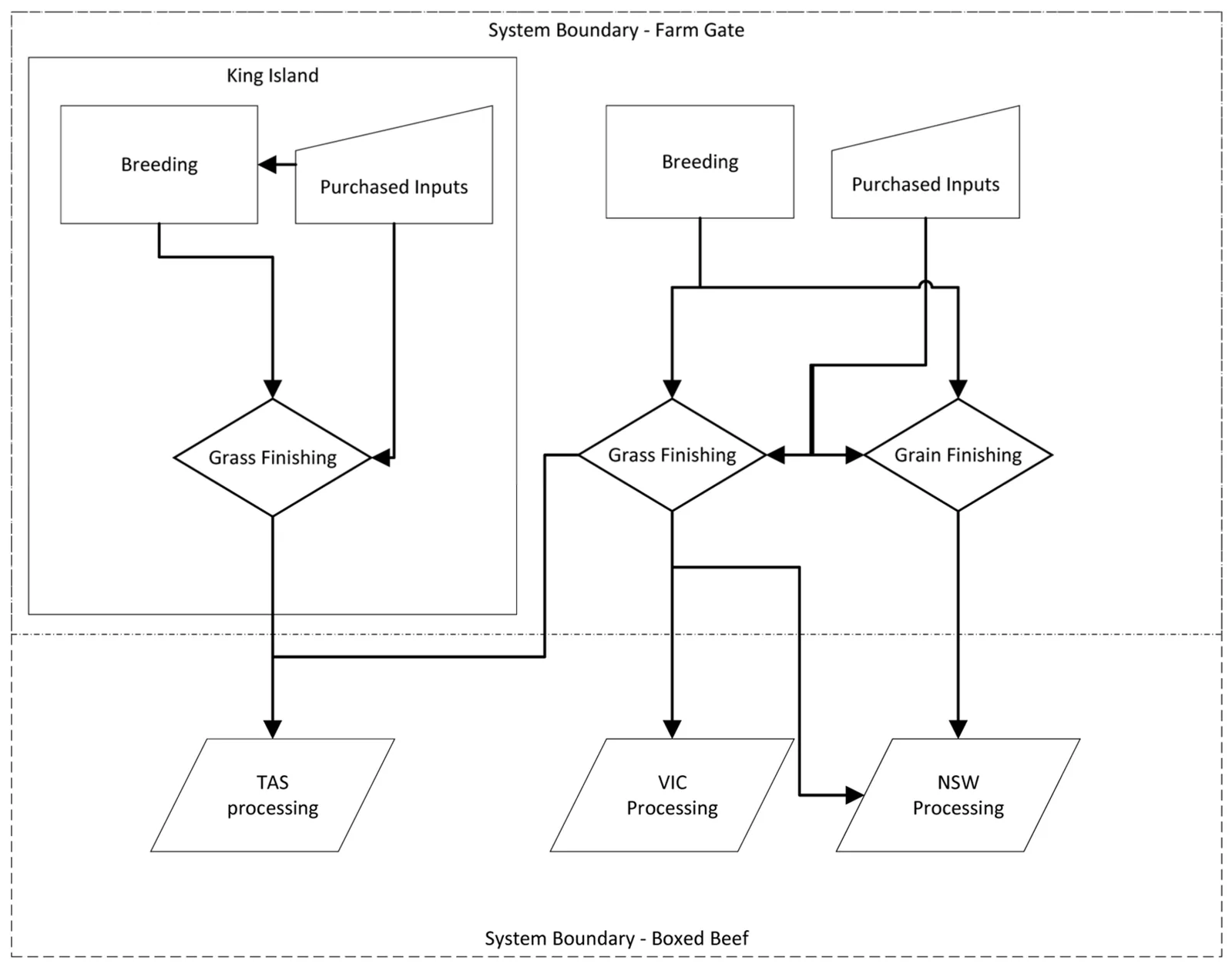
Life cycle assessment system boundaries: (i) farm gate system boundary (dash-dotted line) for all regions and data collection methods; (ii) boxed beef system boundary (dashed line), encompassing the farm gate system boundary and the impacts from meat processing required to generate the reference flow of ‘1 kg of boxed beef’. TAS = Tasmania; VIC = Victoria; NSW = New South Wales.

This study completed a cradle-to-farm-gate and cradle-to-processor-gate product CF using a reference flow of ‘one kg of LW’ on-farm immediately prior to processing, and ‘one kg of boxed beef’ at the exit gate of the processing plant ready to transport to the customer. The product CF used an LCA approach as required in the International Organization for Standardization (ISO) standards ISO 14067, ISO 14040 and ISO 14044 [23,24,25]. LCA is a primary tool for the assessment of environmental impacts and environmental efficiency within supply chains, and typically assesses impacts relative to the functional output of the system (typically, one unit of output, termed the ‘functional unit’, with the corresponding ‘reference flow’ being the amount of product required to fulfil it; see Figure 1). In this study, the functional unit was defined as the delivery of 1 kg of product at each assessed system boundary, with reference flows of 1 kg of LW at the farm gate and 1 kg of boxed beef at the processor gate. Boxed beef was inclusive of all human edible products, which included offal products packed for human consumption. Carbon footprinting, when following the LCA approach, is a comprehensive means of assessing carbon impacts for a product, using a defined system boundary.

The supply chain was supplied by registered farms, located in the southern states of Australia, including New South Wales (NSW), Victoria (VIC), South Australia (SA) and Tasmania (TAS). The system boundary included pre-farm and on-farm emission sources from farm services (e.g., purchased feed, diesel, petrol, electricity, administration) and other purchased inputs (e.g., herbicides and pesticides). Impacts were reported for cattle exiting the production system at the point where cattle were ready for transport for meat processing. Meat processing was then assessed at three processing plants in southern Australia. Impacts were reported for boxed meat leaving the processing plant, reported using a reference unit of ‘one kg of boxed beef’, which was selected to align the product type and chosen end-point of the primary processing system, as recommended by the Livestock Environmental Assessment and Performance (LEAP) Partnership [26]. For clarity, ‘boxed beef’ refers to the chilled or frozen packaged beef leaving the plant; edible offal was retained within the product mass used for allocation (Section 2.5) rather than treated as a separate co-product, so the processor gate functional unit represents the edible product expressed per kg of boxed beef. Meat was segmented into brands within the program based on both production aspects, including region and feeding type, and meat quality. These are described as follows: The King Island (KI) brand was supplied by Farm Assured cattle from King Island, an island located to the south of the Australian mainland. The Portoro brand was supplied by Farm Assured cattle finished on a natural grain ration with no antibiotics or hormones. The Little Joe, Pinnacle and Great Southern brands were supplied by Farm Assured and were based on meat quality attributes. Brand-specific data were provided with the processing data. Each animal was categorised using a market category. Table S1 shows the brands in the study characterised by finishing feed type.

### 2.2. Inventory Data

#### 2.2.1. Farm Gate

Data were collected from 200 of the ~1600 farms that supplied a material (*n* > 30) number of cattle to the supply chain. The surveys from the 200 farms covered 56,336 heads. This dataset represented an 11% sample of the total supply of cattle to the program in the 2020–2023 calendar years. The surveys of supplier farms were conducted between 2020 and 2023, with each survey covering a 12-month production period.

Farms were sampled from a spread of regions (Figure S1, Table S2), with varying productivity and head contribution (Table 1), brands (Table S3), and years (Table S4). The survey questions focused on purchased inputs and herd data. The surveyed farms were sampled from regions represented across the full supply chain (Figure S1). Cattle produced for all markets except Portoro were grass-fed, free-range cattle, with no added antibiotics, hormones or genetically modified organisms (GMOs) in their feed. Cattle produced for the Portoro brand were finished on natural grain [18].

To assess representativeness with respect to supplier size, surveyed farms were compared with the full supply chain frame across annual-supply bins (Table S2). Because larger farms were preferentially enumerated, they are over-represented on a per-farm basis: farms supplying fewer than 75 heads annually comprise approximately 61% of supply chain farms but 40% of the surveyed sample, whereas farms supplying 175 heads or more comprise 6.5% of farms but 22.5% of the sample. On a throughput basis, the surveyed farms account for approximately 19% of the mean annual cattle supply.

The supply chain model was parameterised using regional datasets and methods described by the authors previously [12]. Turnoff from each farm was modelled to reflect the output from a stable herd by removing the impact of changes in inventory, as required by the LEAP guidelines [26]. To determine impacts for cattle from the whole program, in addition to the survey of farms, primary data were obtained from all livestock supplied to the program, which included 514,922 heads for 4 years of throughput. Data available for all cattle processed in the program included information on their weight and age and their region of origin (Figure S1). A description of the parameters used and their sources has been included in Table S5. Merging these two datasets, combined with regional statistics for all the supplier regions, provided a fully characterised model of all cattle and all output from the program. Key parameters from the 200 farms and from the merged dataset representing all farms are shown in Table 1, with further information regarding purchased inputs shown in Table S6 and information about the ration used for cattle processed through the Portoro brand shown in Table S7. Records of slaughter date, dentition, and calving date were used to determine livestock age at point of slaughter.

#### 2.2.2. Meat Processing

Primary data were collected at three processing plants where all cattle were processed. All data related to energy use, waste water management (Table 2), and mass and aggregate economic fractions for product flows through processing (Table 3) were assessed using primary datasets. Impacts were allocated to hides and renderables based on economic value. Retail beef and edible offal together constitute the beef product to which the majority (91%) of processing emissions were allocated; hides, bones and renderables were treated as co-products. Inputs from processing (Table 2) were allocated to products and co-products according to the allocation factors in Table 3.

### 2.3. Greenhouse Gas Emissions Calculations

This study conducted livestock greenhouse gas (GHG) emission modelling using Australian National Greenhouse Gas Inventory (NGGI) methods [27] with state-specific activity data using methods previously described for grazing systems [12] and grain-finishing [17]. Within the NGGI, emissions from grazing cattle are estimated based on relationships between dry matter intake (DMI) and methane production derived from an analysis of Australian respiration chamber data [28]. The estimation of DMI was based on Australian derived relationships that rely primarily on the animals’ weight, weight gain and reproductive status [27]. The methods comply with the international guidelines for conducting livestock LCA [26]. The enteric methane equation of Charmley et al. [28] produced similar methane yields per kg of DMI as the Intergovernmental Panel on Climate Change (IPCC) [29] for grass-fed cattle and was applied for grass-fed cattle. For grain-finished cattle, an enteric methane yield of 13.6 g CH_4_ kg^−1^ DMI from IPCC [29] was applied for cattle finished on natural grain. Further information has been provided in the Supplementary Materials regarding the specific emission calculation methods and factors used for significant emission sources. The study methods were not able to differentiate methane yields between suppliers that grazed cattle on pastures or forages that have recently been shown to reduce enteric methane, such as Chicory [30,31], plantain [32], or forage brassicas [33,34]. Where these pastures were grazed, the study results may underestimate potential methane reductions. No suppliers were identified that used anti-methanogenic supplements such as Bovaer [35,36] or Asparagopsis [37,38]. Further research is needed to provide both inventory data and calculation methods suitable for quantifying these practices in the supply chain.

### 2.4. Land Use and Land Use Change

All states of Australia where the program sourced cattle were net sinks in Australia’s GHG Inventory with respect to forest cover- and soil carbon-related emissions and removals for the years assessed and for the previous 10 years (to 2014) [27]. Regional average data were considered representative of the ~1600 farms supplying the program, and a conservative approach was taken where no emissions or removals were attributed to beef (i.e., zero change in LULUC impacts from soil or vegetation was attributed to beef). Future methodological development is required to assess forest carbon removals and emissions at a supply chain scale using traceable systems in order to confirm that no emissions arise from land use and land use change (LULUC) sources and to identify and quantify removals occurring through tree planting, native regeneration of forests and potentially soil, which are key to reducing the net emissions of Australian beef [39].

### 2.5. Impact Assessment

The study assessed GHG emissions using the IPCC Sixth Assessment Report (AR6) Global Warming Potential (GWP) for a 100-year time horizon of 27 for methane and 273 for nitrous oxide [40]. Greenhouse gas emissions were reported as carbon dioxide equivalents (CO_2_-e). Modelling was conducted with SimaPro™ version 10.2 [41], using an AR6 GWP_100_ characterisation method and a custom beef production model previously published in Wiedemann et al. [12,17,22,42,43]. Background data were sourced from the Australian Life Cycle Inventory Database (AusLCI) [44] where available, or the European ecoinvent Database (3.0) [45].

### 2.6. Handling Co-Production

Farm services and purchased inputs associated with multiple enterprise systems (beef, sheep and cropping) were subdivided, and inputs associated with crop production and sheep were excluded following recommendations from ISO 14044 [25] and as applied in previous studies [12,46]. Impacts were allocated to by-products and co-products of boxed beef and edible offal using the methods reported in Wiedemann and Yan [47] and Wiedemann et al. [12] and included handling edible offal as part of the product mass and allocating to hides and renderables on an economic value basis. Consistent with this, the product mass to which the primary economic allocation was applied (91%; Table 3) comprised retail beef and edible offal combined, with hides, bones and renderables carrying the remaining 9% as co-products. Processing impacts assigned to this product were distributed across the combined edible product mass, such that the reported processor gate result (per kg of boxed beef) reflects the intensity of the edible product rather than boxed beef assessed in isolation.

### 2.7. Uncertainty Analysis

The uncertainty in CF results was analysed for a sample of 200 farm surveys. Stratification of results was initially done ‘per-response’, and each response was analysed based on the progeny status of the cattle: i.e., whether the owner of the livestock directly prior to slaughter also owned the cattle when they were born. This progeny status was classified as the ‘Owner-bred’ status.

The farm gate CF results with primary data were then stratified on year of slaughter, weight at slaughter (to the nearest 10 kg hot standard carcass weight—HSCW) and dentition at slaughter. The purpose of this was to determine the correlation between biophysical factors (dentition, an indicator of age and mass measured as HSCW), and the CF of beef.

To determine the relative uncertainty between primary data CFs and full supply chain CFs, the Mann–Whitney U-test was used. This non-parametric statistical test was selected due to the distributions of the CFs failing to meet the assumptions of normality and homogeneity of variance required for a parametric test. The Mann–Whitney U-test uses sample data to determine if the outcome variable with a non-normal distribution differs across two groups. The whole supply chain result, stratified by brand and processing plant (*n* = 11), was compared with the farm dataset footprints (*n* = 244). The effect size of this difference was measured using Cliff’s delta.

In order to assess the impact of weight and age of cattle on the CF, data were stratified by weight and dentition bins with width 10 kg HSCW and two permanent incisors, respectively, with a CF calculated for each category. This stratification was used to develop a multivariable model which predicted CF as a function of HSCW and dentition at slaughter. The biological nature of cattle production implies that the slaughter weight of an animal results in a hyperbolic change in the CF (with all other variables kept constant) and that a change in the dentition (as a proxy for age) results in a proportional change in the CF. Based on this biological axiom, a base equation was developed to predict the CF from these two carcass characteristics, with the form

(1) $$cf\left(hscw,dentition\right)=\frac{W}{hscw}+D\times dentition+C$$

*W*, *D* and *C* denote unknown constant values, and *HSCW* and *dentition* denote the HSCW and dentition at slaughter, respectively.

**Figure 2:**
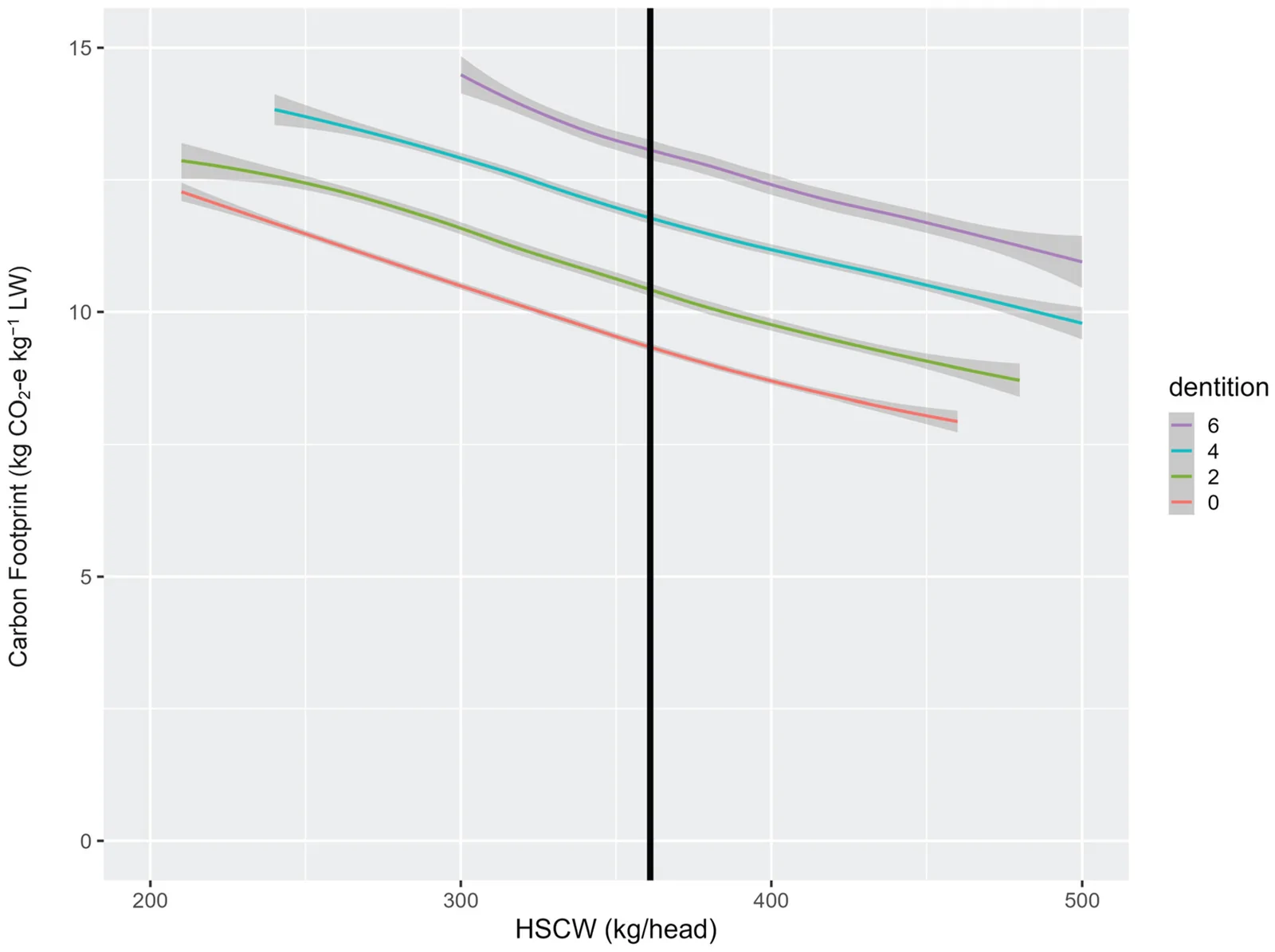
Stratified analysis of CFs from 200 farm surveys showing the relationship between age, weight and CF. The vertical line denotes average carcass weight for sample (361 kg HSCW). CF = carbon footprint; HSCW = hot standard carcass weight.

Using a non-linear least squares curve fitting algorithm, we developed a mechanistic, multivariable model that predicted the CF based on weight at slaughter and dentition at slaughter. This model was trained on a sample of 70% of the data stratified by HSCW bins and dentition (Figure 2). The remaining 30% of the same dataset were used after fitting to validate the model. The model was validated by calculating the root mean square error (RMSE), mean absolute error (MAE) and coefficient of determination (*R*^2^) for the held-out 30% partition of the dataset that were partitioned by HSCW and dentition. The uncertainty in the model coefficients (*W*, *D*, *C*) was quantified by calculating their standard errors and 95% confidence intervals (CIs).

Sensitivity analysis was performed to determine the effect of age-estimation errors on the CFs of animals with the lowest individual animal CFs (CF < 8.5). This was done by creating a transformed probability distribution function (tPDF) of joining window lengths (WL) (i.e., the days between bulls introduced to cows and bulls removed from cows) from the data supplied by farms, weighted on the number of females joined in each window and resampling the final age of each of these low-CF animals. Resampling was achieved by drawing a window length w∈WL from the new tPDF, selecting a conception offset $c\in [-\frac{1}{2}w,\frac{1}{2}w]$ and using the resultant offset c to either add or subtract on-farm days from the animals’ life. The average daily gain of these animals was then adjusted to ensure that the same final slaughter weight was used across all samples within an animal sample set. This changed the estimate of age by 33 days (central 95% of samples, standard deviation 16.8 days). The CF was then re-estimated using LCA and the methods described above. The maximum lower half-width CI and maximum upper half-width CI was calculated to demonstrate the limits of the variation in this lower-bound CF estimate. The sensitivity of the model to uncertainty in the estimate of dressing percentage was assessed using Taylor Series uncertainty in conjunction with the multivariable model developed above. The dressing percentage estimate was based on the information provided in the MLA cattle assessment manual [48], which includes uncertainty ranges of 1–4%.

(2) $$hscw=LW\times dressing_{\%}$$

Substituting Equation (2) into Equation (1) gives

(3) $$cf\left(LW,dressing_{\%},\;dentition\right)=\frac{W}{LW\times dressing_{\%}}+D\times dentition+C$$

Applying Taylor Series uncertainty propagation to Equation (3) with respect to dressing percentage,

(4) $$\sigma_{cf}=\left|\frac{d\;cf}{d\;dressing\%}\right|\times \sigma_{dressing_{\%}}$$

Evaluating the partial derivative analytically,

(5) $$\sigma_{cf}=\frac{W}{LW\times dressing_{\%}^{2}}\times \sigma_{dressing_{\%}}$$

$\sigma_{dressing_{\%}}$ was estimated as the standard deviation of the widest applicable range in dressing percent estimates (4 percentage points, young cattle and heavy steers), assuming a uniform distribution, thus giving 1.2 percentage points. Uncertainties were then calculated for each record of the supplied kill data to obtain a 95% confidence level for the sensitivity of the model to dressing percent uncertainty.

The sensitivity of the supply chain boxed beef CF to impacts from the net change in emissions from the biogenic cycle, representing stock changes in soil and pasture vegetation, and emissions of methane were investigated using Monte Carlo analysis. A total of 1000 iterations per strata were assessed, with only the biogenic parameters (Table S8 [49,50,51,52,53,54,55,56]) varied. This isolates the uncertainty contributed by the biogenic carbon-flow parameters from every other uncertain factor in the model.

Uncertainty associated with model parameters including farm inputs [57] and emission factors (Table S9 [58,59,60,61]) were assessed using Monte Carlo (SimaPro™ version 10.2) [41] analysis, with 1000 iterations per strata for the full supply chain results (11,000 total). This provided a 95% confidence interval for the results. This analysis allowed the presentation of beta (model) uncertainty, as described in Leinonen et al. [62].

## 3. Results

### 3.1. Farm Gate CF

#### 3.1.1. Primary Data

**Figure 3:**
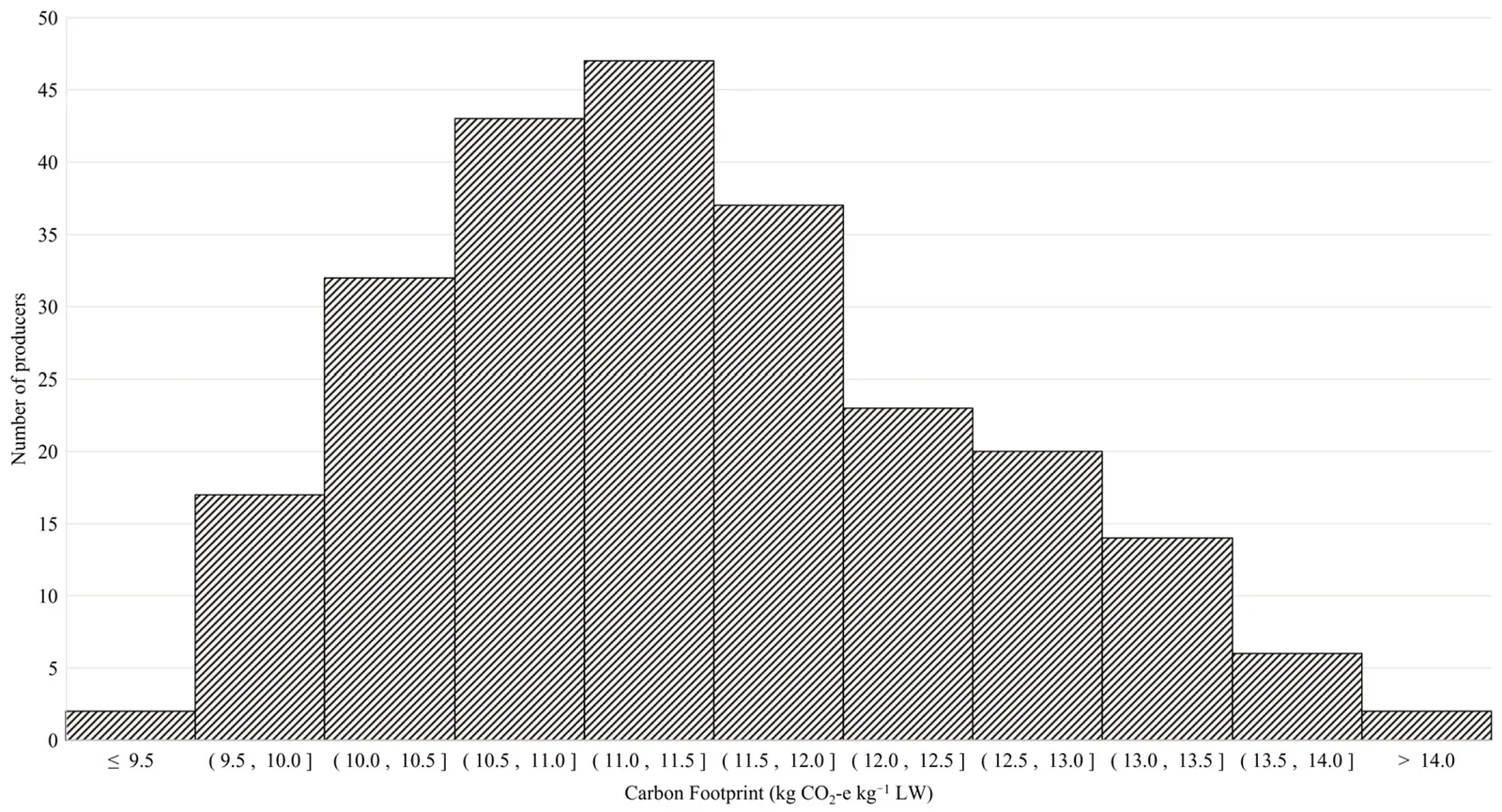
Distribution of carbon footprints (CFs) for 200 farm surveys. Note that >200 CFs are present. This is because farm surveys were stratified on the ‘Owner-bred’ status of the cattle—thus, a single farm may have produced both cattle born on the same farm from which they were consigned to the processor (‘Owner-bred’ = True) and/or cattle which were born on a farm different to that from which they were consigned to the processor (‘Owner-bred’ = False). A single farm may produce both categories of cattle, in which case that farm would have two results.

**Figure 4:**
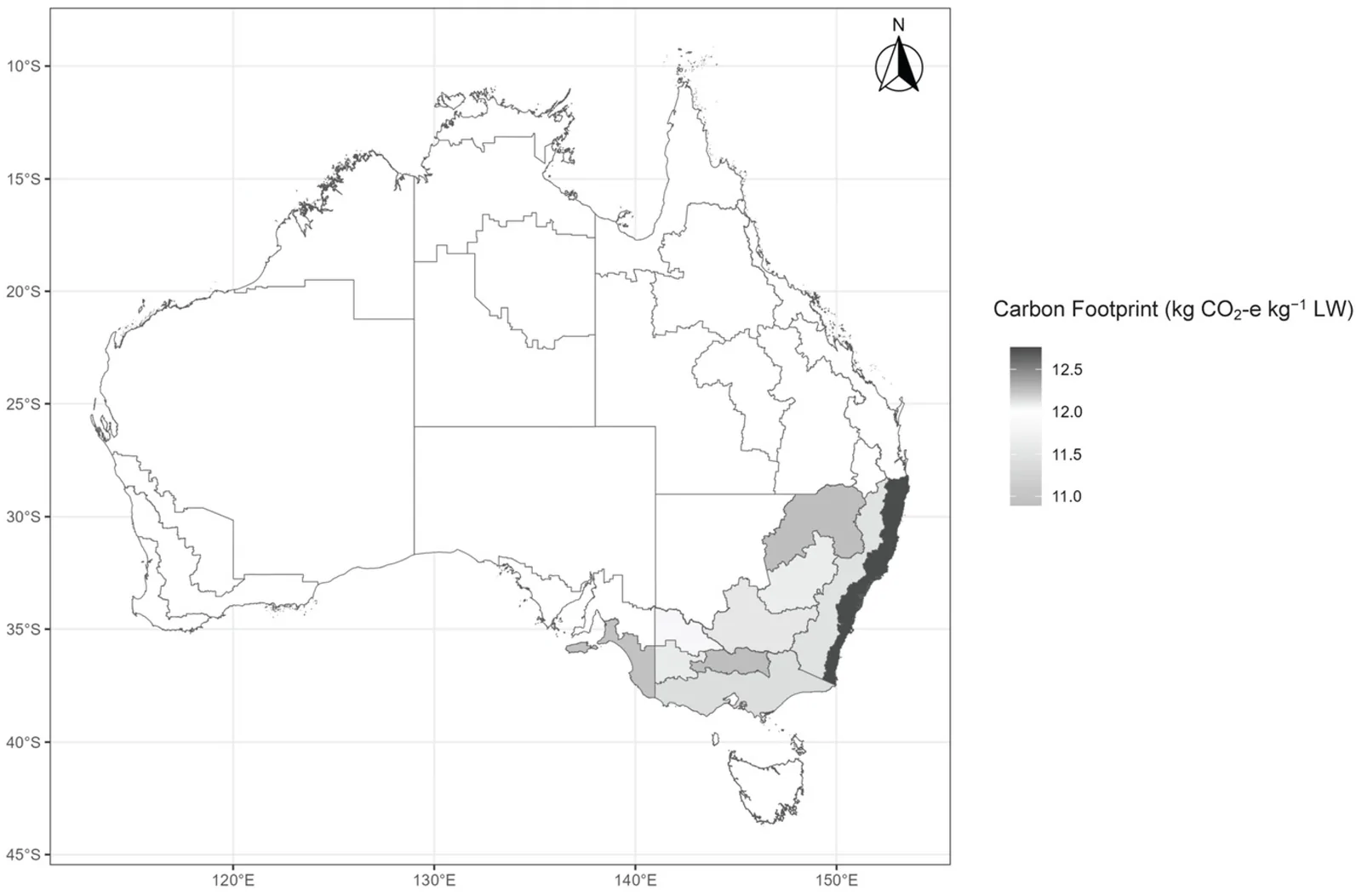
Regional hotspots of CFs from surveyed farms, showing sourcing regions in southern Australia. CF = carbon footprint. Region outlines are aligned with the Australian Agricultural and Grazing Industries Survey (AAGIS) zones and regions [63].

The weighted mean CF (excluding land use and direct land use change-dLUC) from surveyed producers was 11.4 kg CO_2_-e kg^−1^ LW, with a standard deviation of 1.0 kg CO_2_-e kg^−1^ LW (Figure 3). In absolute terms, the farm gate results ranged from 9.1 to 15.0 kg CO_2_-e kg^−1^ LW. Between regions, CFs differed by almost 15%, with the coastal region in NSW having a higher CF (Figure 4).

When data were analysed per year, there was no evident trend, indicating that the farm gate CF had not materially changed in the calendar years 2020–2023.

When the data were stratified and analysed based on individual animal records rather than the total output from each farm, the range in CFs increased, from a maximum of 15.3 kg CO_2_-e kg^−1^ LW to a minimum of 7.9 kg CO_2_-e kg^−1^ LW for grass-finished cattle and 6.1 kg CO_2_-e kg^−1^ LW for grain-finished cattle.

#### 3.1.2. Regionally Parameterised Model

**Figure 5:**
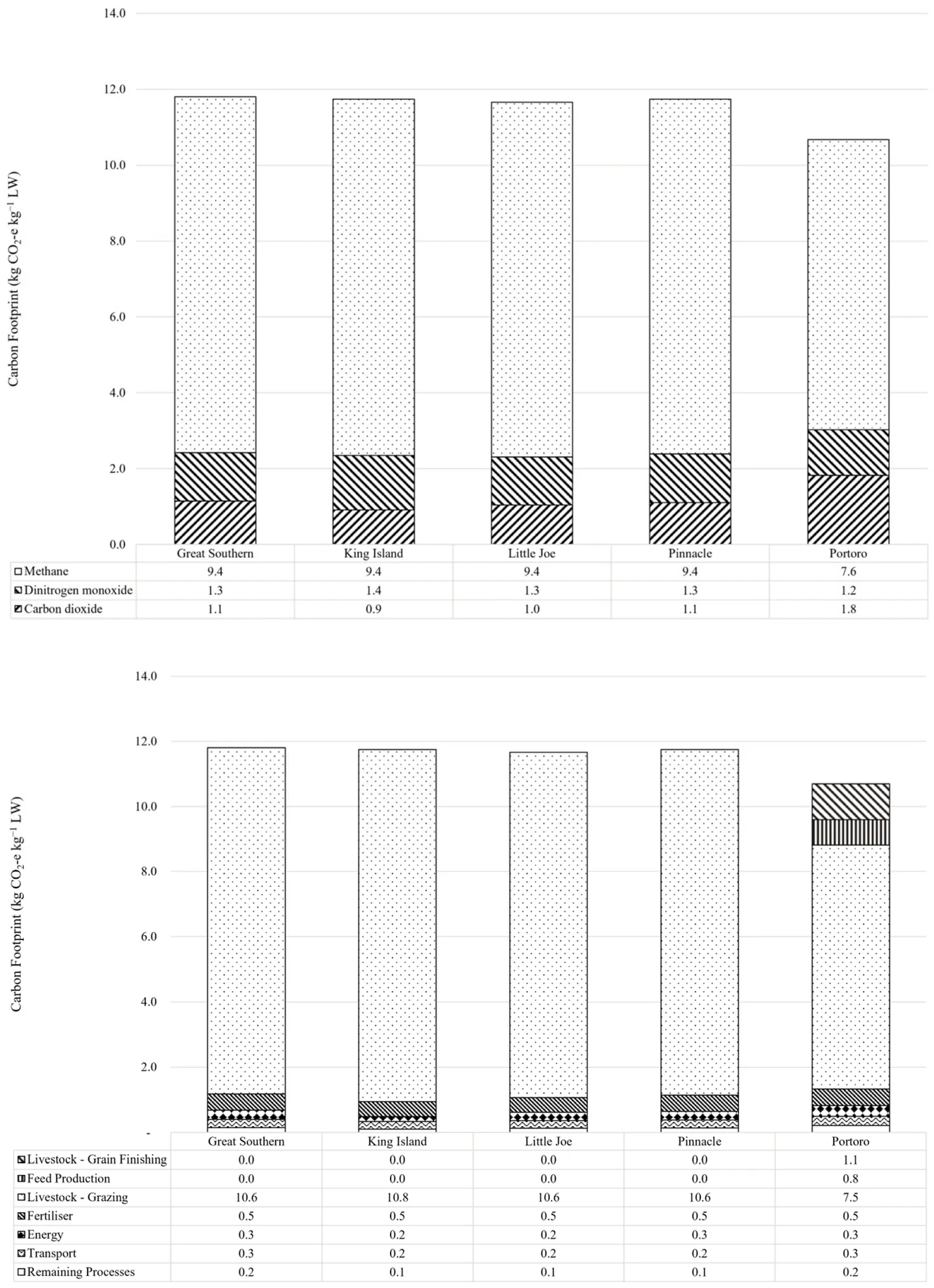
CF (farm stage) of cattle based on the brand and associated brand attributes related to meat quality characteristics. Results were modelled using a regionally parameterised supply chain model. CF = carbon footprint.

Analysis of the whole supply chain for 2023 showed a weighted mean CF of 11.7 kg CO_2_-e kg^−1^ LW (standard deviation 0.4 kg CO_2_-e kg^−1^ LW), with no material difference between grass-finished brands (Figure 5) and a lower CF for grain-finished cattle for the Portoro brand (10.7 kg CO_2_-e kg^−1^ LW, Figure 5).

**Figure 6:**
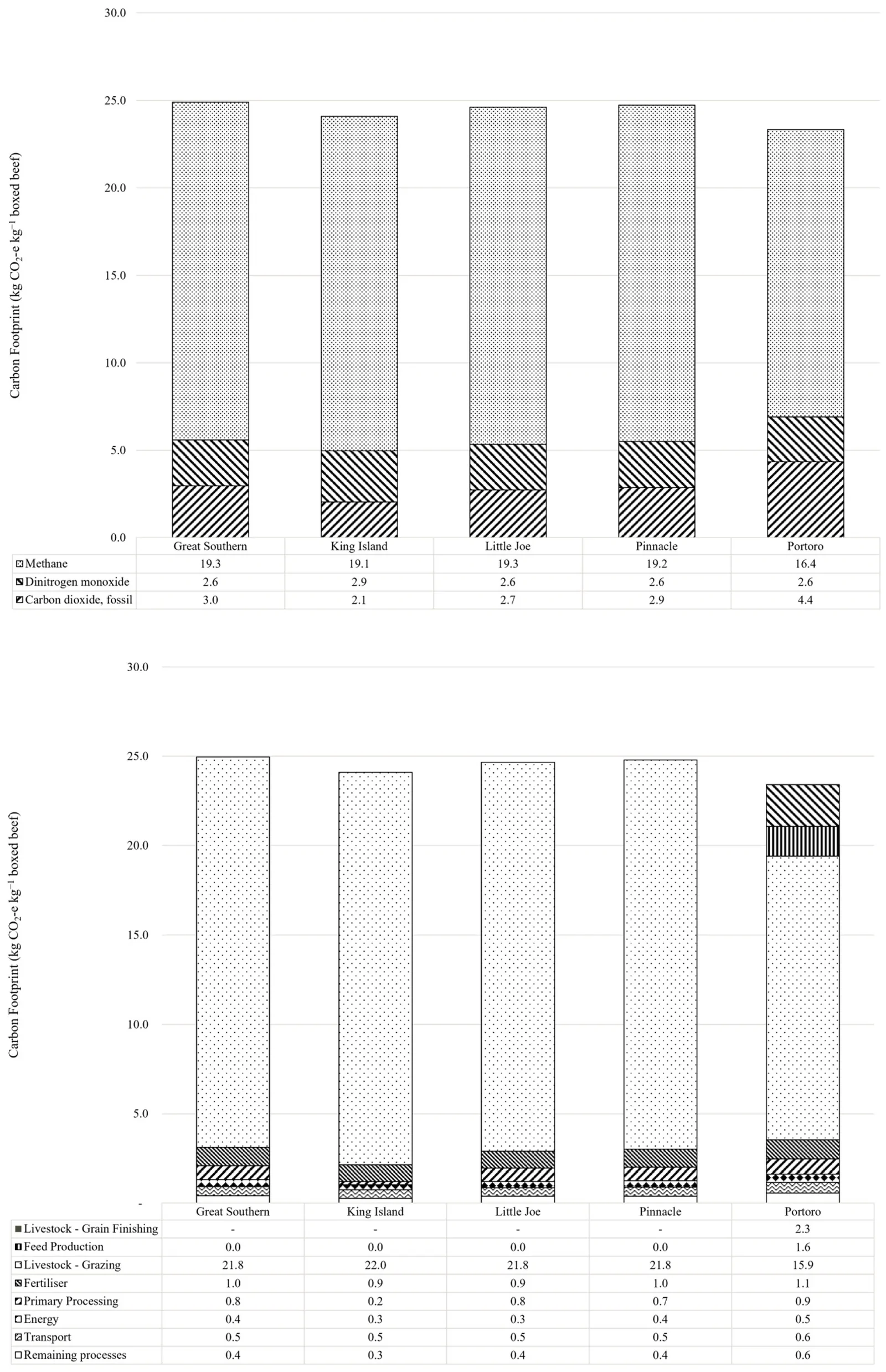
CF of brands (boxed beef). Results were modelled using a regionally parameterised supply chain model. CF = carbon footprint.

The CF for processed beef averaged 24.1 kg CO_2_-e kg^−1^ boxed beef. Results ranged from 23.4 to 24.9 kg CO_2_-e kg^−1^ boxed beef (Figure 6), with a standard deviation of 0.7 kg CO_2_-e kg^−1^ boxed beef.

### 3.2. Uncertainty Analysis

Non-linear least squares fitting of Equation (1) to the 70% training partition (*n* = 220) yielded the model

(6) $$cf\left(hscw,dentition\right)=\frac{1913.6}{hscw}+0.595\times dentition+4.223$$

All three parameters were estimated with high precision and differed significantly from zero (*p* < 0.001). The weight coefficient *W* was 1913.6 (95% CI: 1828.8–1998.5), the dentition coefficient *D* was 0.595 (95% CI: 0.571–0.620), and the constant *C* was 4.223 (95% CI: 3.943–4.502). The sign and form of the fitted coefficients are consistent with the biological axiom underpinning Equation (1).

The model explained the majority of the variation in the stratified CF, with an *R*^2^ of 0.940, RMSE of 0.371, and MAE of 0.286 kg CO_2_-e kg^−1^ LW for the training data. Applied to the held-out 30% validation partition (*n* = 95), which was not used in fitting, predictive performance was retained (*R*^2^ = 0.930), with an RMSE of 0.384 and an MAE of 0.293 kg CO_2_-e kg^−1^ LW.

Sensitivity analysis was performed to determine the effect that the uncertainty in age at slaughter—estimated from calving date, dentition, and kill date—had on the CF of individual animals. The error range in the age of cattle at slaughter was 33 days (95% CI), and the sensitivity analysis showed that the variation in the estimate of the lowest-CF animals (CF < 8.5) was ±0.23 kg CO_2_-e kg^−1^ LW (95% CI). All lowest-CF animals remained in the low-CF tail: none were displaced from the extreme group.

Uncertainty in dressing percentage estimates contributed a mean uncertainty in CF of ±0.23 kg CO_2_-e kg^−1^ LW (95% CI).

When the biogenic flow-model parameter uncertainty was propagated through the whole supply chain CF, the biogenic result varied from 20.0 to 29.0 kg CO_2_-e kg^−1^ boxed beef (95% CI), with a coefficient of variation of 9.4%. There was no difference in the mean of this ensemble compared with the supply chain weighted mean.

When parameter uncertainty was propagated through the regionally parameterised model, the 95% confidence interval was 21.4–27.3 kg CO_2_-e kg^−1^ boxed beef (*n* = 11,000, mean 24.3), with a coefficient of variation of 6.4%. Enteric methane dominated both the mean result and its uncertainty, contributing approximately three-quarters of the total CF. This parameter uncertainty is distinct from, and larger than, the between-brand variation reported above, which describes differences among supplier strata (alpha uncertainty) rather than uncertainty in the underlying factors.

## 4. Discussion

### 4.1. Variation and Emission Reduction Potential

The CF in the Great Southern supply chain was 10% lower than the national average of 12.7 kg CO_2_-e kg^−1^ [64], and the CF of the surveyed farm dataset was 15% lower than the national average. While variation in CF driven by differences in the production system is established [13], there is less insight into variation within production systems and almost no knowledge regarding the variability at the individual animal level. This study found the lowest CF at the farm scale was 29% below the supply chain average, while individual animals were identified that had impacts 48% below average, with the lower bound approaching a biologically constrained minimum CF. This lower bound is constrained by the minimum amount of methane and manure emissions that can be generated based on the biophysical limits of beef cattle herds, viz, the number of cows, bulls and replacement females required to produce each calf, and the minimum amount of time required for a young animal to grow to market weight. In the present study, the modelled minimum for these regions was effectively 9.5 kg CO_2_-e kg^−1^ LW, with only 1% of producers having impacts below this in the reporting years (see Figure 3).

The extreme limits of the range reflected a small number of individual animals that represented the absolute lowest and highest weight-for-age performance within the dataset. These results may be slightly over- or under-estimated because the age estimate for each animal was based on the farm-average calving date rather than a calving date specific to each animal. It is possible that the best performing animals with respect to weight-for-age were the oldest in their cohort, while the worst performing cattle may have been younger in their cohort. The sensitivity analysis results show that the impact of this effect is limited to 5% of the mean supply chain CF, demonstrating that the peak performance of the absolute best animals in the cohort is materially lower than both the lowest farm-average CF and the supply chain mean CF. With this caveat noted, the results demonstrate peak performance outcomes and provide targets for genetic progress.

These results suggest that CFs at farm gate had a practical minimum in the order of 9–10 kg CO_2_-e kg^−1^ LW, which could be seen as a target for improvement. Furthermore, individual animals may achieve CFs as low as 6 kg CO_2_-e kg^−1^ LW, setting lower targets for what may be achieved through the most productive animals. The most measurable trend at the supply chain scale was the growth rate and finished weight leading to lower lifetime emissions. This points to the opportunity to reduce emissions through productivity measures. However, further research is needed to understand the potential trade-offs that may occur in programs optimised for growth and carcass weight. Maintaining moderate cow weight, which is important for whole-herd feed efficiency, is one expected challenge because of the relationship between a higher mature cow weight and a higher growth rate [65]. With this noted, these results suggest that improved production systems may be able to deliver a further 15–20% reduction in the CF of grass-finished beef below the levels reported here. Further reductions could then be achieved through the feeding of low methane pastures, forages [66] and supplements [67] and through removals achieved by improved soil and vegetation management on farms.

Variations between regions in the regional analysis were driven by cattle production efficiency, namely the biological performance of the herd, measured in terms of weaning rates and young cattle growth rates. The coastal region of NSW had a higher carbon footprint, when compared to others, due to the grazing conditions available. The north-western region of NSW showed slightly elevated CFs which corresponded to higher inputs in this region. This was related to the prevalence of fodder cropping and other practices that elevated impacts from fossil fuel energy inputs and fertiliser.

### 4.2. Supply Chain Model

In order to test the robustness of the supply chain model, impacts were compared between CFs calculated from the surveyed dataset and CFs from those same farms, calculated only using the regional model, where primary data were only from attributes that could be determined at processing, viz, the age and weight of the animal and the region where each animal was bred. The CF was found to be 5% lower when analysed using farm survey data compared to when analysed using only data available from processing. This could either indicate that the supply chain model was systematically conservative or that the surveyed farms had slightly lower impacts than the regional average datasets which were used in the supply chain model. Farms analysed using primary data had lower use of inputs, particularly diesel and electricity, than predicted using the regionalised model.

Results were also compared to explore the differences between the weighted average CF for the 200-farm survey dataset with the supply chain average (excluding Portoro). The Mann–Whitney U-test determined that the results were numerically lower but not statistically different (*p* = 0.15) in the farm survey dataset compared to the supply chain model. The effect size was small (Cliff’s *δ* = −0.19, 95% CI −0.36 to 0.00). As previously noted, this result could either indicate that the regionally parameterised model was systematically conservative or that the farms which supplied primary data had better herd performance and input use efficiency than other farms in their region, and the average performance of other farms in those regions genuinely produced beef with a higher CF than those farms that provided primary data.

The multivariable model revealed a strong relationship between carcass weight, dentition, and carbon footprint (Figure 2). This suggests that heavier, younger animals at slaughter are associated with lower emissions per unit of beef produced, highlighting the potential for targeted genetic and management interventions to reduce supply chain emissions. This model is separate to the regionally parameterised supply chain model described above and was primarily used to understand the core carcass characteristics that drive the LW CF of cattle production.

### 4.3. Boxed Beef Results

The CF of boxed beef, when categorised by market, was highly correlated with the CF (LW; *R*^2^ = 0.996), but only when the KI brand was removed. The CFs of boxed products were between 2 and 2.1 times higher than their associated LW footprints because most of the impacts were allocated to edible meat, which accounted for less than 50% of the LW mass. A small amount of emissions were added in the processing phase, mostly from energy use. In Tasmania, where the KI was processed, the electricity grid was largely powered by hydroelectric energy, which had a very low CF [27]. Consequently, the KI CF (boxed) had a lower impact relative to its LW CF when compared with the other market categories. Despite the significant focus on supply chain impacts from beef, few large-scale studies using primary data have been published world-wide, resulting in few comparison studies for the boxed meat results showing the relationship between production system specifics and results. For context, the average impacts reported for boxed beef were lower than the values reported for boxed meat produced using average production systems in the United States (US) of 31.3 kg CO_2_-e kg^−1^ meat [68] when converted to the same reference unit. This is an area where further research and studies relying on large, traceable datasets will improve market-level insights. Furthermore, with large individual animal datasets, new insights will develop for marketing lower-emission products that have been identified through harnessing the best production practices and performance.

### 4.4. Land Use and Land Use Change

**Figure 7:**
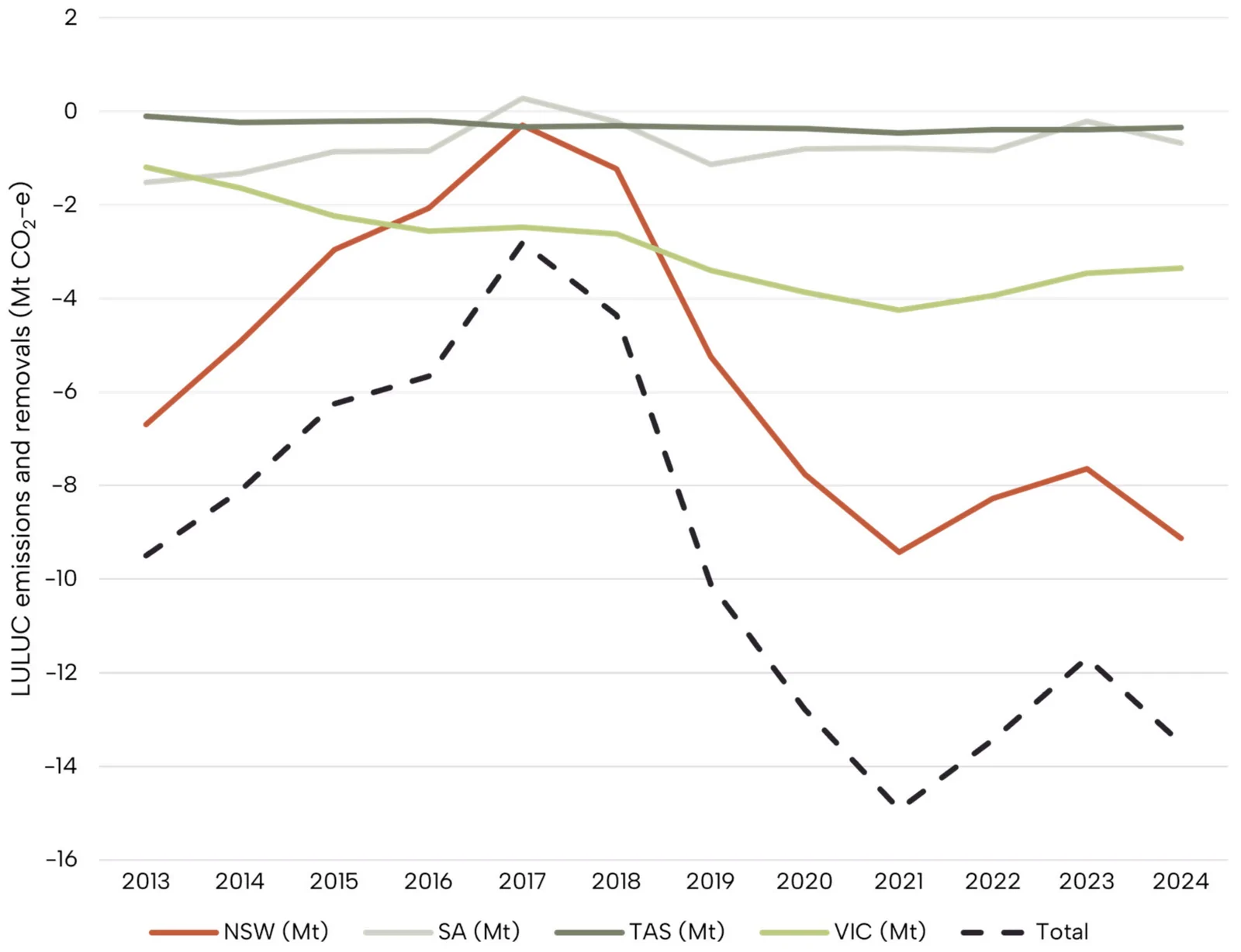
Annual LULUC emissions and removals (Mt CO_2_-e) as reported by Australia’s National Greenhouse Accounts, Sector: 4.C.1.ii Grassland soils [69]. LULUC = land use and land use change; Mt = megatonnes; NSW = New South Wales; SA = South Australia; TAS = Tasmania; VIC = Victoria.

Modelled biogenic flows had no net impact on the supply chain CF. This is a conservative implementation of Australia’s National Greenhouse Accounts data [69] (Figure 7), which showed that grassland soils in the relevant states were net sinks in the study period.

**Figure 8:**
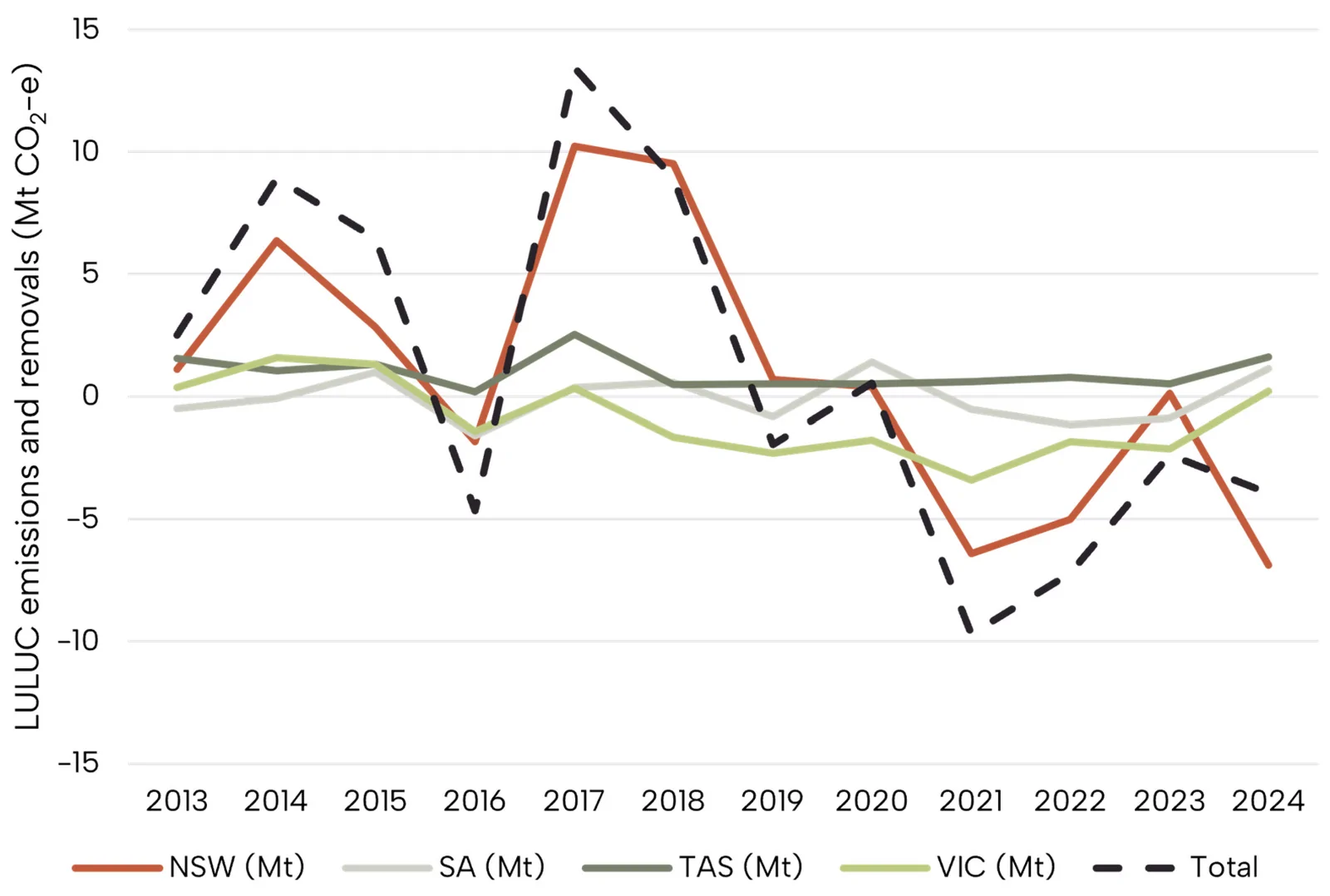
Annual LULUC emissions and removals, as reported by Australia’s National Greenhouse Accounts, Sector: 4.C Grassland [69]. LULUC = land use and land use change; Mt = megatonnes; NSW = New South Wales; SA = South Australia; TAS = Tasmania; VIC = Victoria.

**Figure 9:**
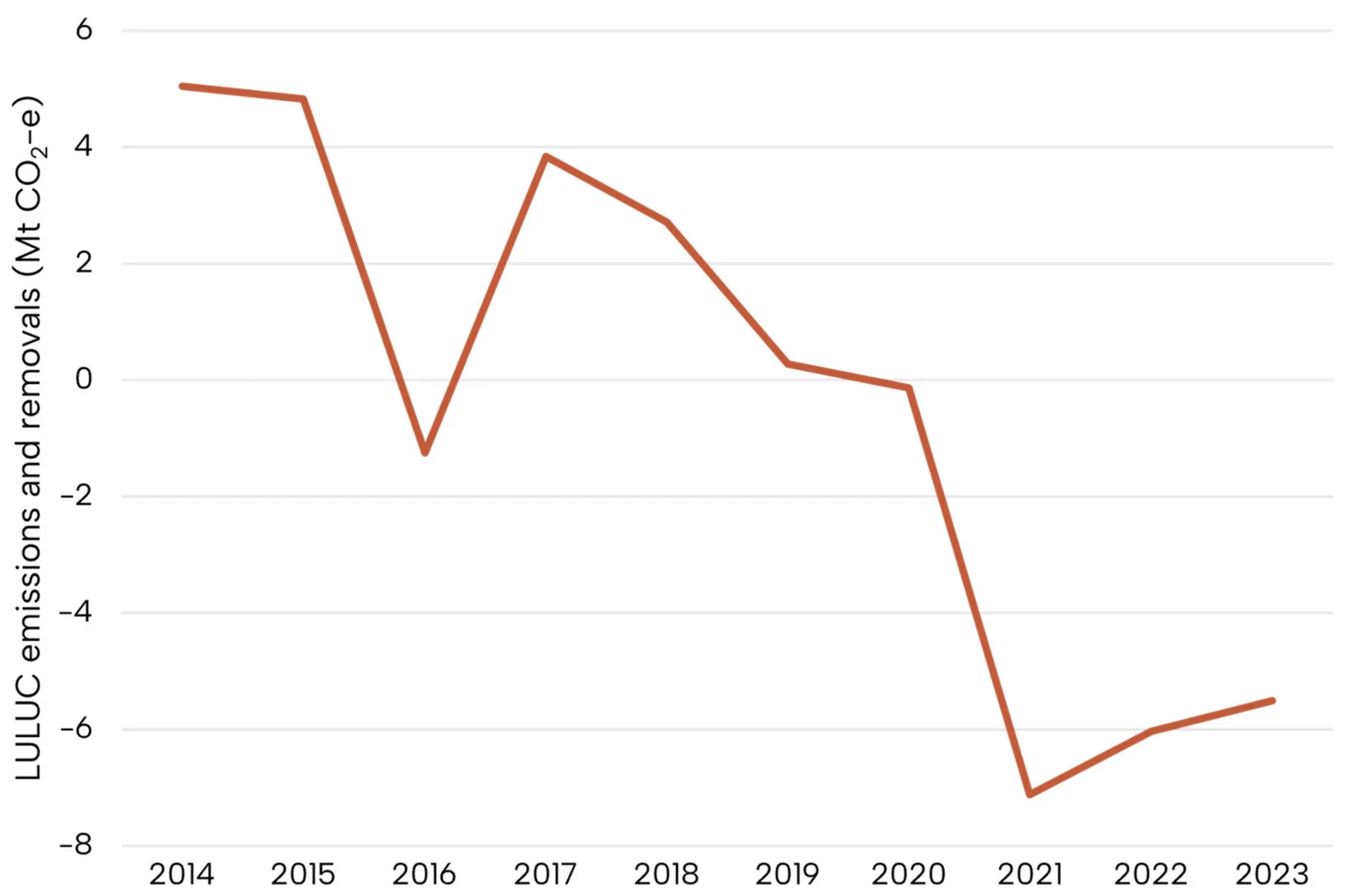
LULUC total emissions for Australian states from which the supply chain drew material numbers of cattle. Values are linearly discounted LULUC emissions and removals over a 20-year discounting period (Mt CO_2_-e). LULUC = land use and land use change; Mt = megatonnes.

Systems producing cattle from well-managed pastures may remove emissions through soil and trees on farms [70], but verification of this relationship remains challenging, and some published studies have not considered the permanence of soil carbon storage [71]. Because of these challenges with verification, the most conservative approach was taken in the present study (assuming zero removals), which was supported by regional LULUC reporting in the Australian GHG Inventory [27]. The published national beef estimate [72] ranges from +2.6 to −1.1 kg CO_2_-e kg^−1^ LW depending on whether a 20- or 10-year linear period is applied—a range that brackets zero. Sensitivity is presented as ±2.6 kg CO_2_-e kg^−1^ LW for the whole supply chain result, corresponding to the larger absolute bound. This is conservative for the supply regions in this study: the underlying state-level results show LULUC as a net removal for cattle in NSW, Victoria and South Australia under 20-year linear discounting, with the positive national figure driven by Queensland and the Northern Territory (Figure 8 and Figure 9). To enable the reporting of removals, verifiable, supply chain-scale measurement capability is urgently needed, and future work should aim to develop supply chain-level quantification of soil and vegetation carbon to address this knowledge gap.

### 4.5. Environmental and Carcass Performance

The combination of a large-scale dataset with individual animal records for weight and age at processing provided new insights into the peak performance possible from grass-finished cattle, and enabled a comparison with carcass quality attributes. The present study demonstrated that CF continued to decline with carcass weight and reached the minimum level for cattle with 0 and 2 permanent incisors at carcass weights of 400 kg HSCW and a minimum level for cattle with 4–6 permanent incisors at carcass weights of 500 kg HSCW. The inverse relationship between LW gain (weight-for-age) and CF is consistent with the regression relationship derived in Wiedemann et al. [12]. However, in that previous study, finished weight did not show a defined relationship with CF, as was found here. This was most likely because the difference in weight and weight gain was not as large as in Wiedemann et al. [12], where the dataset was not available on an individual animal basis, making it impossible to disaggregate the weight-for-age trend from the finished weight trend, and where CF could not be stratified by dentition. One reason why higher-weight cattle at reasonably young ages achieved better outcomes in the present study was because achieving heavy carcass weights improved whole-herd efficiency, i.e., maximum beef per cow unit. Heavier carcasses from animals closer to mature age and weight also allowed for a fuller expression of marbling, since intramuscular fat accumulates as a proportion of muscle only after muscle maturity is approached and there is sufficient time for the muscle to ‘fill up’ with fat [73,74]. The present analysis revealed that the CF of high-marbling brands (Little Joe) was equal to the performance of the supply chain average while delivering better meat quality outcomes. This was a positive finding, considering that this brand sourced cattle only from grass-finished cattle. Further research is needed to explore the peak performance of breeding herds for both meat quality and CF performance both in grass and grain finishing systems.

## 5. Conclusions

The CF of cattle in a large supply chain varies significantly between farms and animals. The lowest farm-average and individual animal CFs were 29% and 48% lower than the supply chain average, respectively. This variation is predictable from HSCW and dentition at slaughter, suggesting that supply chain-scale modelling may be used to identify beef with lower-than-average carbon footprints. The study presented results for a boxed beef brand, supplied from approximately 1600 Farm Assured suppliers and 130,000 heads per annum of cattle to premium grass and natural grain brands, representing a large-scale analysis using traceable datasets from farm production to boxed product. Future research should be conducted to determine how to best leverage this information to reduce supply chain carbon footprints through improved production performance.

Boxed beef and LW CFs were lower in the present study than previous Australian research [72] and were lower than the average of beef produced in major trading nations such as the US when the respective CFs were analysed using country-specific methods. This demonstrated that grass- and natural grain-finished beef could be produced with comparably low CFs.

## Figures and Tables

**Table 1:** Key production and productivity data for herds supplying the Farm Assured program.

Parameter	All Farms ^1^	Surveyed Farms ^2^
Mean slaughter weight (kg LW)	588	617
Dentition at slaughter	2.1	2.0
Cow weight (kg)	637	639
Weaning rate (%)	89%	90%
Age at slaughter (days)	749	739
Mean lifetime average daily gain (kg per day)	0.75	0.79
Heads supplied	514,922	55,384

^1^ Measured parameters from processing plant (slaughter weight, dentition, head number) and modelled parameters based on regional weighted averages for cow weight and weaning rate. Age at slaughter and lifetime average daily gain were defined in the model, derived from primary data. ^2^ Measured parameters from primary data. LW = liveweight.

**Table 2:** Energy inputs used at three processing facilities reported per tonne of hot standard carcass weight (HSCW).

Input	Units (Per Tonne HSCW)	Amount (Weighted Average)
Electricity	kWh	438.69
Liquefied petroleum gas (LPG)	L	0.22
Natural gas	MJ	2255.61
Diesel	L	0.63
Fuel oil	MJ	0.00
Petrol	L	0.07

**Table 3:** Mass and economic allocation factors used to determine product flows and emissions allocation in processing products, by-products and co-products (primary data).

Product	Mass (kg per Head)	Economic Allocation
Retail beef and edible offal	264.5	91%
Hides	n.r.	1%
Bones	n.r.	2%
Renderables	n.r.	6%
Total		100%

n.r. = not reported.

## Data Availability

The data presented in this study are not publicly available due to commercial sensitivity.

## Supplementary Materials

The following supporting information can be downloaded at: https://www.mdpi.com/article/10.3390/ani16162498/s1, Table S1: Characterisation of products in the JBS Southern Australia Farm Assured program by cattle feed type; Figure S1: Regional distribution of whole supply chain (left) and survey farms (right) across southern Australia; Table S2: Regional distribution of whole supply chain and survey farms within the supply chain; Table S3: Distribution of supply chain, characterised by brand and data source (surveyed, non-surveyed); Table S4: Distribution of supply chain, characterised by year and data source; Table S5: Description of modelled and primary parameters used in the supply chain model; Table S6: Farm purchased input use normalised to one tonne of dry matter intake and key herd and farm parameters; Table S7: Ration composition for cattle processed through the Portoro natural grain-finished brand; Table S8: Assumptions used in the modelling of biogenic carbon flows; Table S9: Assumptions used in propagating uncertainty in the beef model results.

## Abbreviations

The following abbreviations are used in this manuscript:

AR6	IPCC Sixth Assessment Report
AusLCI	Australian Life Cycle Inventory database
CF	Carbon footprint
CO_2_-e	Carbon dioxide equivalent
dLUC	Direct land use change
DMI	Dry matter intake
FA	Farm Assured
FLAG	Forest, Land and Agriculture
GHG	Greenhouse gas
GLEAM	Global Livestock Environmental Assessment Model
GMO	Genetically modified organism
GS	Great Southern
GWP	Global Warming Potential
HSCW	Hot standard carcass weight
IPCC	Intergovernmental Panel on Climate Change
ISO	International Organization for Standardization
JBS	JBS Southern Australia Pty Ltd.
KI	King Island
LCA	Life cycle assessment
LEAP	Livestock Environmental Assessment and Performance
LPG	Liquefied petroleum gas
LULUC	Land use and land use change
LW	Liveweight
MAE	Mean absolute error
NGGI	National Greenhouse Gas Inventory
NSW	New South Wales
RMSE	Root mean square error
SA	South Australia
SBTi	Science Based Targets initiative
TAS	Tasmania
tPDF	Transformed probability distribution function
US	United States
VIC	Victoria
