# Supplementary material for: Multivariable Analysis of the Carbon Footprint of a Branded Beef Supply Chain Using Individual Animal Data and Carcass Characteristics
Source: Animals (Basel). 2026 Aug 11;16(16):2498. doi: 10.3390/ani16162498 (PMC13508965; doi:10.3390/ani16162498)
Supplement: Supplementary file 1 [file animals-16-02498-s001.zip › animals-4421400-supplementary.pdf]

Supplementary Material for the manuscript titled ‘Multivariable Analysis of the Carbon Footprint of a Branded Beef Supply Chain Using Individual Animal Data and Carcass Characteristics’.

O’Shannessy and Wiedemann, 2026.

Table S1. Characterisation of products in the JBS Southern Australia Farm Assured program by cattle feed type

Brand	Includes grass	Includes natural grain
Great Southern	Yes	No
King Island	Yes	No
Little Joe	Yes	No
Pinnacle	Yes	No
Portoro	No	Yes

Note: grass = pasture-finished, no grain or grain by-products, no added antibiotics, hormones, or GMOs in feed; natural grain = grain-finished without antibiotics or ionophores.

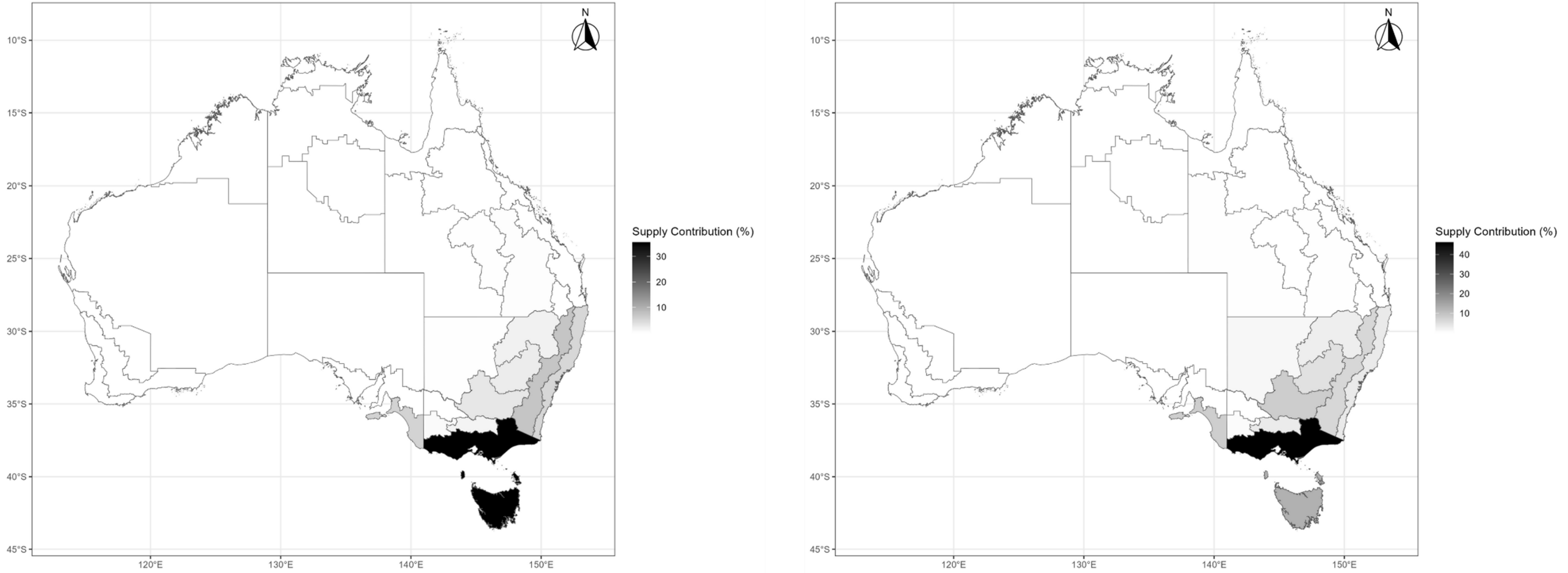

Figure S1. Regional distribution of whole supply chain (left) and survey farms (right) across southern Australia

Note: Shading indicates supply contribution (%).

Table S2. Regional distribution of whole supply chain and survey farms within the supply chain

State and Region	Non-surveyed	Surveyed	Total
**New South Wales**			
Central West	2%	5%	3%
Coastal	5%	4%	5%
Far West	0%	1%	0%
North West Slopes and Plains	2%	3%	2%
Riverina	3%	8%	4%
Tablelands (Northern, Central and Southern)	7%	7%	7%
**South Australia**			
Murray Lands and Yorke Peninsula	0%	0%	0%
North Pastoral	0%	0%	0%
South East	6%	8%	6%
**Tasmania**			
Tasmania	36%	13%	33%
**Victoria**			
Central North	2%	3%	2%
Mallee	0%	0%	0%
Southern and Eastern Victoria	36%	47%	37%
Wimmera	1%	1%	1%
**Total**	**100%**	**100%**	**100%**

Table S3. Distribution of supply chain, characterised by brand and data source (surveyed, non-surveyed)

Brand	Non-surveyed	Surveyed
Great Southern	95%	5%
King Island	87%	13%
Little Joe	94%	6%
Pinnacle	94%	6%

Table S4. Distribution of supply chain, characterised by year and data source

Year	Non-surveyed	Surveyed
2020	30%	8%
2021	24%	9%
2022	24%	16%
2023	23%	67%

Table S5. Description of modelled and primary parameters used in the supply chain model

Supply chain stage and parameter description	Primary data	Regional estimate	Mix of primary and regional
**Breeding**			
Breeding cow weighted mean days on feed, including culled animals	No	No	Yes
Breeding mortality rate	No	No	Yes
Bull average daily gain	No	Yes	No
Bull cull rate	No	Yes	No
Bull days on farm	No	Yes	No
Bull inclusion rate	No	Yes	No
Calf average daily gain birth to weaning	No	Yes	No
Calf days birth to weaning	No	Yes	No
Cow cull rate	No	No	Yes
Cow head number	No	No	Yes
Cow weight in	No	No	Yes
Cow weight out	No	No	Yes
Crude protein of pasture	No	Yes	No
Dry matter digestibility of pasture	No	Yes	No
Dry matter of faeces	No	Yes	No
Gain in pregnant cows	No	No	Yes
Milk adjustment factor	No	Yes	No

Supply chain stage and parameter description	Primary data	Regional estimate	Mix of primary and regional
Milk consumed (calves)	No	Yes	No
Milk production	No	Yes	No
Replacement bull average daily gain	No	Yes	No
Replacement bull days on feed	No	Yes	No
Replacement heifer average daily gain weaning to joining	No	No	Yes
Replacement heifer days from weaning to joining	No	No	Yes
Standard reference weights	No	Yes	No
Weaning rate	No	No	Yes
Weaning weight	No	Yes	No
Finishing (grass)	No	No	No
Crude protein of pasture	No	Yes	No
Dry matter digestibility of pasture	No	Yes	No
Dry matter of faeces	No	Yes	No
Standard reference weights	No	Yes	No
Young cattle days weaning to slaughter	No	No	Yes
Young cattle growth rate	No	No	Yes
Young cattle head number	No	No	Yes
Young cattle slaughter age	No	No	Yes
Finishing (natural grain)	No	No	No
Dry matter digestibility of natural grain ration	Yes	No	No
Feed intake of cattle fed natural grain ration	Yes	No	No

Supply chain stage and parameter description	Primary data	Regional estimate	Mix of primary and regional
Manure management	Yes	No	No
Ration ingredients	Yes	No	No
Weaning weight	Yes	No	No
Young cattle mortality rate	Yes	No	No
Meat processing	No	No	No
Allocation factors	Yes	No	No
Mass flows	Yes	No	No
Meat processing energy use	Yes	No	No
Meat processing waste water	Yes	No	No
Scope 3 other (accounting, advisory, vet, repairs and maintenance, etc.,)	Yes	No	No
On-farm (general)	No	No	No
Leaching fraction	No	Yes	No
Mean temperature	No	Yes	No
Pasture wet fraction	No	Yes	No
Scope 3 other (accounting, advisory, vet, repairs and maintenance, etc.,)	No	Yes	No
Services (fuel, fertiliser, etc.,)	No	No	Yes
Wet fraction	No	Yes	No

Table S6. Farm purchased input use normalised to one tonne of dry matter intake, and key herd and farm parameters

Input	Mean	Min	Max	Standard deviation
Diesel (litres/tonne DMI)	1.760	0.000	18.902	2.870
Petrol (litres/tonne DMI)	0.364	0.000	9.181	0.923
Electricity (kWh/tonne DMI)	4.038	0.000	195.989	18.000
Diammonium phosphate (tonnes/tonne DMI)	0.001	0.000	0.016	0.002
Lime (tonnes/tonne DMI)	0.026	0.000	0.682	0.081
Monoammonium phosphate (tonnes/tonne DMI)	0.000	0.000	0.007	0.001
Other fertiliser (tonnes/tonne DMI)	0.019	0.000	0.862	0.097
Singe superphosphate (tonnes/tonne DMI)	0.009	0.000	0.104	0.015
Urea (tonnes/tonne DMI)	0.002	0.000	0.044	0.006
**Herd parameters**				
Cows joined (head)	1,000	20	17,420	1,604
**Farm parameters**				
Production area (ha)[1]	969.8	40.1	9,537.5	1,790.7

[1] Farm areas were only available for a subset of 35 of the 200 farm surveys.
DMI = dry matter intake; ha = hectares.

Table S7. Ration composition for cattle processed through the Portoro natural grain-finished brand

Ingredient	Ration contribution
Barley	65%
Canola	8%
Other	2%
Hay	4%
Straw	4%
Silage	7%
Hulls	8%
Molasses	3%
**Total**	**100%**

Table S8. Assumptions used in the modelling of biogenic carbon flows

Parameter	Value	Uncertainty	Distribution type	Reference
Pasture utilization rate (%)	35	± 14.3%	Triangle	Johnson et al. [49]
Root:shoot ratio	4.224	± 25%	Triangle	Mokany et al. [50]
Pasture respiration rate (%)	50	± 10%	Triangle	Parton et al. [51]
Carbon content in dry pasture biomass (% of dry matter)	44.73	± 10%	Triangle	Ma et al. [52]
Carbon content in protein (% of protein)	53	N.A.	-	Rouwenhorst et al. [53]
Carbon content in fat (% of fat)	75	N.A.	-	Bray & Bouchard [54]
Carbon in manure (% of volatile solids)	55	± 10%	Triangle	Natural Resources Conservation Service [55]
Soil respiration rate and organic matter turnover (% of freshly deposited organic material respired)	100	± 1%	Triangle	Value based on soil carbon stock change potential determined from the IPCC [56]

N.A. = not applicable

Table S9. Assumptions used in propagating uncertainty in the beef model results

Emission source	Key parameters	Uncertainty	Distribution type	Reference
Beef faecal N_2O EF		50%	Triangle	Grazing beef - from National Greenhouse Accounts Factors [58]
Beef NH_3 EF		20%	Triangle	Grazing beef - from National Greenhouse Accounts Factors [58]
Beef urine N_2O EF		50%	Triangle	Grazing beef - from National Greenhouse Accounts Factors [58]
Enteric methane	20.7 g CH_4 kg $^{-1}$ DMI	1%	Triangle	Charmley et al. 2016 [28]
Feed intake	kg DMI head^{-1} day^{-1}	20%	Uniform	Applied for beef cattle from Poppi [59]
Fertiliser N_2O EF		36%	Uniform	Australian Crop Factor - National Inventory Report [60]
Indirect N_2O EF		50%	Uniform	De Klein et al. [61]
Manure CH_4 uncertainty		20%	Triangle	Uncertainty of +/-20% - National Greenhouse Accounts Factors [58]
Manure N_2O uncertainty		50%	Triangle	Uncertainty of +/-50% - National Greenhouse Accounts Factors [58]
Manure NH_3 uncertainty		20%	Triangle	Uncertainty of +/-20% - National Greenhouse Accounts Factors [58]
N_2O leach runoff EF		50%	Triangle	De Klein et al. [61]

EF = emission factor; DMI = dry matter intake
